# Gender-affirming care education in pharmacy: a scoping review protocol of practices in Canada and the USA

**DOI:** 10.1136/bmjopen-2024-086927

**Published:** 2025-01-02

**Authors:** Liam Jackman, Rakhshan Kamran

**Affiliations:** 1 Temerty Faculty of Medicine, University of Toronto, Toronto, Ontario, Canada; 2 Nuffield Department of Orthopaedics, Rheumatology and Musculoskeletal Sciences, University of Oxford, Oxford, UK

**Keywords:** Review, Health Equity, Health Education, Health Services for Transgender Persons, MEDICAL EDUCATION & TRAINING

## Abstract

**Abstract:**

**Introduction:**

Gender-affirming care refers to a range of social, psychological, medical and/or surgical interventions provided to affirm one’s gender. Pharmacists play a key role in gender-affirming care and are involved with choosing optimal treatments, monitoring progress/side-effects and providing education. However, it is currently unknown what gender-affirming care education is provided to pharmacy students and pharmacists according to information available in published literature. This is important to identify potential education gaps in pharmacy curricula and an understanding of the current state of gender-affirming care education in pharmacy.

**Methods and analysis:**

This scoping review protocol follows guidance from Arksey and O’Malley and Levac and colleagues. The population, concept and context framework are used to specify the research question and subsequently the search strategy. Database searching will occur across four databases: Medline, Embase, APA PsycINFO and Scopus; with the search date ranging from inception to 1 January 2025. Eligible studies will include pharmacy students or pharmacists, and examine the presence of gender-affirming care pharmacy education in Canada and the USA. There are no restrictions to date. Article screening and extraction will occur independently and in duplicate. Educational interventions, gender-affirming care content and recommendations extracted from each article will be narratively synthesised. This work is undertaken with patients and public involvement.

**Ethics and dissemination:**

Ethical approval is not required as this is a review of published studies and does not collect any human data. Results will be disseminated through a peer-reviewed publication and conference presentations.

**STRENGTHS AND LIMITATIONS OF THIS STUDY**

- This scoping review utilises a comprehensive search strategy, informed by patient and public partners and evidence-based LGBTQ+search filters.
- Articles eligible for inclusion in this scoping review are limited to English and French and published in Canada or the USA; therefore, it is possible that relevant studies in other languages/countries may be missed.
- The scoping review aims to capture a comprehensive picture of gender-affirming care education in pharmacy, but current practices may not always be published in the literature; therefore, it may not reflect the breadth of practices within pharmacy education.

## Introduction

 For most, gender is congruous with the sex assigned at birth. For some, however, gender is incongruous with the sex assigned at birth (transgender and gender diverse persons).^1^ Gender dysphoria, psychological distress stemming from the incongruence between one’s gender and sex assigned at birth can occur.^2^ Gender-affirming care includes social, psychological, medical and/or surgical interventions to affirm one’s gender.^3^ Gender-affirming care also includes practices that demonstrate awareness of and respect for different gender identities, including asking about and using a person’s name and pronouns, adopting gender-neutral language, and using a bioanatomy-based approach (which considers a person’s anatomy without making assumptions about gender identity).^4 5^ These practices are pivotal and though seemingly small, can have big impact on patient experiences, patient outcomes and quality of life.^5^ Gender-affirming care affirms one’s gender, alleviates gender dysphoria and augments well-being. Gender-affirming care promotes positive identity development, self-esteem and social participation, supporting individuals in living true to themselves and forming meaningful connections within their communities.^6^,^10^ Gender-affirming care also results in reduction in anxiety, depression and suicidality.^8 10^

In Canada, the most recent national census identified over 100 000 transgender and non-binary individuals, representing a significant number of people who would benefit from access to gender-affirming care.^1^ Unfortunately, many of these individuals are not able to access it. We know from one large Canadian study that only about half of transgender and non-binary individuals reported receiving gender-affirming care while the other half reported an unmet healthcare need.^11^ Health inequities: disparities in the distribution of health resources and health status, may be attributed in part to cisnormativity in healthcare, the assumption that people will identify with the gender binary (male or female). This bias is perpetuated by educational curricula that exclude or even erase the needs of transgender and non-binary individuals, ultimately resulting in healthcare professionals feeling unprepared to provide competent and culturally responsive care to this population.^12 13^

The Canadian Council for Accreditation of Pharmacy Programmes states that ‘pharmacy graduates must have a broad understanding of health, the factors that contribute to a healthy community including the social determinants of health, and the structure and role of the healthcare and public health systems’.^14^ In order to fulfil this criterion in a way that is inclusive of transgender and gender diverse people, pharmacy curricula should promote awareness of health disparities faced by this population, promote equitable access to healthcare services for this population, and provide teaching of the best practices related to transgender health. In addition, pharmacy curricula must move one step further: not only teaching what care should be delivered but also how to deliver that care in a way that is patient-centred and responsive to the needs of transgender and gender diverse individuals. This requires integrating frameworks of cultural humility and cultural safety. Cultural humility provides students with opportunities for self-reflection, teaching them about the identification of implicit biases, and promoting lifelong learning. Cultural safety must be built on this by addressing the systemic inequities in healthcare, teaching students to critically think about and challenging the structural barriers that contribute to health inequities.^15^

The purpose of this scoping review is to identify the current state of gender-affirming care education in pharmacy, from undergraduate curricula for pharmacy students to continuing education for practicing pharmacists in Canada and the USA. The scoping review aims to identify the content, delivery methods, and learning outcomes of educational initiatives. The scoping review will also explore how cultural humility and cultural safety are incorporated into the existing curricula.

The focus on pharmacy education was deliberate as published literature has suggested that pharmacists’ involvement in gender-affirming care can be beneficial for patients, considering their accessibility to both patients and other healthcare professionals and their expertise in choosing optimal treatments, monitoring progress and/or side-effects, and providing education.^16^ The specific focus on gender-affirming care content distinguishes this article from previous articles that have more generally focused on sexual and gender minority content. This decision was made thoughtfully, acknowledging that transgender and gender diverse individuals have distinct healthcare experiences and health needs that deserve focused attention. Thus, our objective is to provide a more nuanced exploration of this specific area within pharmacy education.

This work offers specific insight into gender-affirming care content in pharmacy education and is complementary to other works, such as ‘Gender-affirming care in undergraduate nursing education’ by Crawford *et al* which offers specific insight into gender-affirming care content in nursing education.^17 18^ These works can contribute to broader discussions on how different healthcare disciplines integrate gender-affirming care education, offering insights into potential areas for interdisciplinary learning and collaboration.

## Methods and analysis

The research design, a scoping review, was deemed most appropriate in light of its ability to provide a comprehensive overview of a wide range of literature, including both empirical research and non-empirical sources (eg, reports, policy documents, educational resources—such as webinars, online modules or online pamphlets). The use of a scoping review also allows for a more exploratory approach, which aligns itself well with the broad nature of our research question. The proposed start and end dates are from 1 January 2025 to 1 January 2026. This scoping review will also facilitate the identification of patterns, trends, and gaps in the literature, providing justification for further research as is increasingly required by granting agencies and journals alike. This scoping review follows the six stages identified by Arksey and O’Malley^19^ and Levac *et al*.^20^ The scoping review will be reported following the Preferred Reporting Items for Systematic Reviews and Meta-Analyses extension for Scoping Reviews (PRISMA-ScR).^21^

### Stage 1: specifying the research questions

The population, concept and context (PCC) framework is being used to specify the research question and subsequently the search strategy.^22^ The population is pharmacy students, pharmacists, faculty, curricula or programmes. The concept is gender-affirming care content, defined as any educational content and/or pedagogical strategy meant to equip pharmacy students and/or pharmacists with knowledge and/or skills necessary to provide culturally safe care to transgender and gender diverse people. The context is Canada and the USA, selected due to the similarity of pharmacy education and practice.

The objective of this scoping review is to identify the current state of gender-affirming care education in pharmacy.

#### Primary research question

- What gender-affirming care education is provided to pharmacy students and/or pharmacists according to information available in published literature?

#### Secondary research questions

- What are the key learning outcomes related to gender-affirming care that initiatives aim to achieve according to the information available in the published literature?
- What are the perceptions of pharmacy faculty and undergraduate students regarding the integration of gender-affirming care education in pharmacy programmes according to the information available in published literature?
- How are gender-affirming care education initiatives delivered?
- How do gender-affirming care education initiatives incorporate principles of cultural humility and safety?

### Stage 2: identify relevant studies

The search strategy was developed in consultation with the research team (first and senior authors, patient and public partners; search strategies available in online supplemental file 1). The search strategy was reviewed by a health sciences librarian with experience in scoping reviews. The search will be conducted using four databases on 1 January 2025: Medline, Embase, APA PyschINFO, and Scopus, with supplementary searches in professional pharmacy association websites to ensure comprehensive coverage of both peer-reviewed and grey literature. There will be no restrictions on the year of publication.

The keywords and subject headings were chosen through collective input from research team members, consideration of keywords and subject headings included in previous works related to gender-affirming care by our team,^23 24^ and consideration of keywords and subject headings in a published population search filter.^25^

#### Inclusion criteria

- Population: Pharmacy students, pharmacists, faculty, curriculum (undergraduate or graduate), and/or schools (including continuing education/employee training).
- Concept: Studies that examine the presence of gender-affirming care education within pharmacy.
- Context: Canada or the USA; articles that focus on transgender or gender-diverse individuals or where a relevant subgroup is available for extraction and analyses.
- Date range: Unrestricted to capture historical and present context.
- Language: English or French, recognising the bilingual nature of Canadian academia, as the official languages of Canada are English and French; further the author team includes individuals fluent in French, therefore screening and extraction in French is feasible.

#### Exclusion criteria

- Population: Non-pharmacy stakeholder.
- Concept: Studies that do not examine gender-affirming care education.
- Context: Study not from Canada or the USA.
- Language: Non-English or non-French.

The search strategy includes terms across the 2SLGBTQ+acronym; however, the focus of this scoping review is on transgender and gender diverse population and gender-affirming care. The articles will be carefully screened in line with the above inclusion/exclusion criteria to include studies which focus on gender-affirming care for transgender or gender diverse populations, or where a relevant subgroup is clearly defined and data available for extraction and analysis.

### Stage 3: select studies

The articles from each database will be exported to EndNote, where a team-member will conduct an initial deduplication. The articles will then be exported to the web-based, literature review management tool, Covidence, to screen search results. The titles and abstracts will be screened by two independent reviewers, with an aim to reach a consensus. The reviewers will pilot test 20 titles and abstracts and then attend a meeting to discuss discrepancies before proceeding further. If consensus cannot be reached, discrepancies will be resolved by a third independent reviewer. Similarly, the full text documents will subsequently be screened by two independent reviewers. The discrepancies will again be resolved by a third independent reviewer.

### Stage 4: chart the data

The data will be extracted by two independent reviewers, with a third reviewer resolving any discrepancies. The data extraction form will encompass bibliographical information such as title, author and year of publication; study design, describing the methodological approach such as qualitative, quantitative or mixed methods; geographic information, specifying the country, province or state and specifying the population; institution; intervention details, including the nature and content of the intervention; and any consideration of cultural humility, and safety within the intervention; outcome measures, such as learning outcomes, assessment methods, student/faculty perceptions; and reported results; theoretical frameworks; barriers and facilitators; key findings, implications and any other ancillary notes. This is a scoping review; therefore, a risk of bias assessment is not required, nor will it be conducted.^26^

### Stage 5: collate, summarise and report results (data analysis)

The findings will be presented in a tabular format, showcasing key aspects such as the target population (students, pharmacists, faculty, curricula, programmes), geographical locations, educational intervention, gender-affirming care content and recommendations extracted from each article. The rationale for this is so that data is displayed in a structured manner, allowing for more effective and efficient comparisons across studies, and facilitating the identification of patterns, trends and gaps. The use of a tabular format does find a limitation in the potential oversimplification of data and omission of important qualitative details.^27^ To address this limitation, we will provide narrative summaries of the included articles.

The data will be analysed using a descriptive-analytical approach, which aligns itself with the overarching aim of a scoping review: to provide a descriptive overview of studies. This will encompass an examination of the prevalence and characteristics of gender-affirming care education within eligible studies. The use of a descriptive-analytical approach allows for the identification of systemic patterns related to educational content, education delivery and educational needs, but does find a limitation in that it may fail to capture smaller, study-specific details and differences.^28^

The results will be conveyed through narrative synthesis, delving into educational content and education delivery with an examination of its impact on pharmacy practice and patient care. The use of narrative synthesis allows for complex information to be synthesised into a comprehensive narrative that captures the core of the research findings but does find a limitation in that it is inherently subjective, relying on individual interpretation of the data, and therefore can lack transparency.^29^ To address this limitation, we will adhere to the synthesis without meta-analysis (SWiM) guidelines to ensure systematic and evidence-based reporting.^29^

### Positionality and patient and public involvement

The authors of the scoping review consist of cisgender and non-binary individuals, including individuals who are members of the 2SLGBTQ+community. All of the authors hold higher-level clinical and research degrees (eg, PhD, MD, PharmD, MSc) and are clinician-scientists. The authors are published researchers in the field of gender-affirming care, with the senior author having completed a doctoral thesis in that area. Seven patients and public partners are also present on the research team. These include three trans women, two trans men and two non-binary individuals. Patients and public partners are predominantly white individuals (6/7). They were recruited through contacting transgender charities (ie, Gender Identity Research and Education Society), online community transgender support groups, and recruitment of one individual from an existing patient group with a separate research team. Patients and public partners are involved in confirming the relevance and importance of the research questions, reviewing the search strategy, and reviewing the final results of the scoping review and manuscript. Patients and public partners will be present as co-authors or mentioned in the acknowledgement section of the final paper, given the level of involvement and if they meet authorship criteria. Patients and public partners are not research subjects but integral members of the research team. Meetings with patients and public partners, as well as other research team members, are conducted via Microsoft Teams, with agendas circulated in advance. For this project and others involving the patients and public partners, an average of 2–3 meetings are held annually, supplemented by regular email communication between meetings. During discussions, any disagreement among patients and public involvement (PPI) members are resolved through discussion until a consensus is reached.

## Ethics and dissemination

### Ethics

This is a scoping review of the literature and there is no primary data collection from human subjects. Therefore, ethics approval is not required. However, ethical consideration include the respectful representation of information from the literature and respect for gender-diversity through word choice and content presentation.

### Dissemination

The findings of this scoping review aim to inform pharmacy students, pharmacists, pharmacy educators, educational institutions, healthcare policymakers, accreditation bodies and professional bodies offering continuing professional development (CPD) about gender diversity and gender-affirming care, specifically augmenting awareness around gender-affirming care education. The findings may be particularly relevant to pharmacy educators and educational institutions who may be able to compare their gender-affirming care educational content and delivery methods to those identified in this review, thereby identifying areas of strength and potential areas for improvement. The findings may also be particularly relevant to healthcare policymakers and accreditation/professional bodies who, in striving to promote inclusive education, may make policy decisions and/or adjust accreditation standards to increase competency.

The findings of this scoping review will be disseminated to the aforementioned stakeholders by the research team through conference presentations (eg, at the World Professional Association for Transgender Health and Rainbow Health Ontario conferences), peer-reviewed publications (eg, in BMJ Open), development of webinars and online resources, and by engaging patient and public partners.

### Implications

It is through understanding the current state of gender-affirming care education in pharmacy that we can identify educational gaps. This can provide a foundation for programme development that is more inclusive of transgender and gender diverse people. Key findings from this scoping review can also help to inform more inclusive health outcome measurement education for gender-affirming care, an area identified as requiring improved healthcare professional knowledge.^2330^,^32^ This has the potential to augment the competence and confidence of future and current pharmacists in providing culturally safe care to transgender and gender diverse individuals, which is critical in a healthcare landscape that often marginalises this population. The study’s findings could also inform policy at multiple levels, encouraging the prioritisation of diversity as a core component of healthcare training. This, in turn, could lead to broader systemic changes in the healthcare system, promoting a more equitable environment for transgender and gender diverse people.

This scoping review protocol outlines a research study which will be conducted to better understand gender-affirming care education in pharmacy. The results of this research can be used by clinicians, policymakers, educators, and commissioners to better identify gender-affirming education in pharmacy, and understand important gaps for improvement. The results from this study will also complement other important gender-affirming care education research conducted across various clinical areas, to ensure comprehensive and holistic progress in gender-affirming care education across various clinical professions and settings.^17 33 34^

### Limitations

The scoping review is limited to English and French; therefore, it is possible that relevant studies in other languages may be missed. The scoping review also aims to capture a comprehensive picture of gender-affirming care education in pharmacy, but current practices may not always be published in the literature, therefore, it may not reflect the breadth of practices within pharmacy education.

## supplementary material

10.1136/bmjopen-2024-086927 online supplemental file 1

## References

[1] Government of Canada SC (2022). The daily — canada is the first country to provide census data on transgender and non-binary people. https://www150.statcan.gc.ca/n1/daily-quotidien/220427/dq220427b-eng.htm.

[2] Fraser L, Trombetta C, Liguori G, Bertolotto M (2015). Management of Gender Dysphoria: A Multidisciplinary Approach.

[3] Lee JY, Rosenthal SM (2023). Gender-Affirming Care of Transgender and Gender-Diverse Youth: Current Concepts. Annu Rev Med.

[4] Waryold JM, Kornahrens A (2020). Decreasing Barriers to Sexual Health in the Lesbian, Gay, Bisexual, Transgender, and Queer Community. Nurs Clin North Am.

[5] Bhatt N, Cannella J, Gentile JP (2022). Gender-affirming Care for Transgender Patients. Innov Clin Neurosci.

[6] Chen D, Berona J, Chan Y-M (2023). Psychosocial Functioning in Transgender Youth after 2 Years of Hormones. N Engl J Med.

[7] Sansfaçon AP, Medico D, Suerich-Gulick F (2020). 'I knew that I wasn’t cis, I knew that, but I didn’t know exactly': Gender identity development, expression and affirmation in youth who access gender affirming medical care. Int J Transgender Health.

[8] Nguyen HB, Chavez AM, Lipner E (2018). Gender-Affirming Hormone Use in Transgender Individuals: Impact on Behavioral Health and Cognition. Curr Psychiatry Rep.

[9] Goetz TG, Arcomano AC (2023). 'Coming Home to My Body': A Qualitative Exploration of Gender-Affirming Care-Seeking and Mental Health. J Gay Lesbian Ment Health.

[10] Green AE, DeChants JP, Price MN (2022). Association of Gender-Affirming Hormone Therapy With Depression, Thoughts of Suicide, and Attempted Suicide Among Transgender and Nonbinary Youth. J Adolesc Health.

[11] Scheim AI, Coleman T, Lachowsky N (2021). Health care access among transgender and nonbinary people in Canada, 2019: a cross-sectional survey. CMAJ Open.

[12] Bauer GR, Hammond R, Travers R (2009). 'I don’t think this is theoretical; this is our lives': how erasure impacts health care for transgender people. J Assoc Nurses AIDS Care.

[13] Crawford J, Schultz A, Chernomas WM (2024). Interpersonal Transphobia Within Nursing: A Critical Concept Exploration. ANS Adv Nurs Sci.

[14] The Canadian Council for Accreditation of Pharmacy Programs (2023). Accreditation standards for canadian educational programs leading to the doctor of pharmacy (pharm.d.) degree. https://ccapp.ca/wp-content/uploads/2023/09/2023-Pharmacy-Stds.pdf.

[15] Public Health Agency of Canada Common definitions on culural safety. Chief public health officer health professional forum. https://www.canada.ca/content/dam/hc-sc/documents/services/publications/health-system-services/chief-public-health-officer-health-professional-forum-common-definitions-cultural-safety/definitions-en2.pdf.

[16] Bishop BM (2015). Pharmacotherapy Considerations in the Management of Transgender Patients: A Brief Review. Pharmacotherapy.

[17] Crawford J, Brandt A, Kramer M (2024). Gender inclusive and affirming practices across undergraduate nursing curriculum: A scoping review. Nurse Educ Today.

[18] Crawford J, Schultz ASH, Linton J (2023). Gender-affirming care in undergraduate nursing education: a scoping review protocol. BMJ Open.

[19] Arksey H, O’Malley L (2005). Scoping studies: towards a methodological framework. Int J Soc Res Methodol.

[20] Levac D, Colquhoun H, O’Brien KK (2010). Scoping studies: advancing the methodology. Impl Sci.

[21] Tricco AC, Lillie E, Zarin W (2018). PRISMA Extension for Scoping Reviews (PRISMA-ScR): Checklist and Explanation. Ann Intern Med.

[22] Peters MDJ, Godfrey C, McInerney P (2022). Best practice guidance and reporting items for the development of scoping review protocols. JBI Evid Synth.

[23] Kamran R, Jackman L, Chan C (2023). Implementation of Patient-Reported Outcome Measures for Gender-Affirming Care Worldwide: A Systematic Review. JAMA Netw Open.

[24] Jackman L, Chan C, Jacklin C (2024). Patient-reported outcome measures for paediatric gender-affirming care: A systematic review. Paediatr Child Health.

[25] Carrie Price MLS (2022). LGBTQIA+. https://carrieprice78.github.io/guides/search-filters/lgbtqia/.

[26] Khalil H, Peters MD, Tricco AC (2021). Conducting high quality scoping reviews-challenges and solutions. J Clin Epidemiol.

[27] Vedantu (2024.). Tabular presentation of data - objectives, parts, illustration, forms and limitations. https://www.vedantu.com/commerce/tabular-presentation-of-data.

[28] Careerfoundry (2022). What is descriptive analytics? A complete guide. https://careerfoundry.com/en/blog/data-analytics/descriptive-analytics/.

[29] Campbell M, McKenzie JE, Sowden A (2020). Synthesis without meta-analysis (SWiM) in systematic reviews: reporting guideline. BMJ.

[30] Kamran R, Jackman L, Laws A (2023). Patient and healthcare professional perspectives on implementing patient-reported outcome measures in gender-affirming care: a qualitative study. BMJ Open Qual.

[31] Kamran R, Jackman L, Laws A (2024). Patient and healthcare professional perspectives on the Practical Guide to Implementing PROMs in Gender-Affirming Care (PG-PROM-GAC): analysis of open-ended responses from patients and healthcare professionals. BMJ Open Qual.

[32] Kamran R, Jackman L, Laws A (2024). Developing feasible and acceptable strategies for integrating the use of patient-reported outcome measures (PROMs) in gender-affirming care: An implementation study. PLoS ONE.

[33] Kamran R, Chan C, Jackman VA (2024). Transgender and Gender Diverse Medical Education in Radiology: A Systematic Review. Acad Radiol.

[34] Dubin SN, Nolan IT, Streed CG (2018). Transgender health care: improving medical students’ and residents’ training and awareness. Adv Med Educ Pract.

