# Supplementary material for: Gender-affirming care education in pharmacy: a scoping review protocol of practices in Canada and the USA
Source: BMJ Open. 2025 Jan 2;15(1):e086927. doi: 10.1136/bmjopen-2024-086927 (PMC11748774; doi:10.1136/bmjopen-2024-086927)
Supplement: online supplemental file 1 [file bmjopen-15-1-s001.docx]

Supplementary File 1. Search Strategy

**Ovid MEDLINE: Epub Ahead of Print, In-Process & Other Non-Indexed Citations, Ovid MEDLINE® Daily and Ovid MEDLINE® <1946-Present>**

1 ("exp Health Services for Transgender Persons/" or "exp Gender Identity/" or "Gender ident*" or "exp Transgender Persons/" or "transgender*" or "transperson*" or "transpeople*" or "trans gender*" or "exp Transsexualism/" or "trans patient" or "trans health*" or "trans* affirm" or "transexual*" or "trans sexual*" or "transman" or "transmen" or "trans man" or "trans men" or "transwoman" or "transwomen" or "trans woman" or "trans women" or "trans masc*" or "transmasc*" or "trans fem*" or "transfem*" or "gender quest*" or "gender fluid*" or "genderfluid*" or "gender non-conforming" or “gender continuum” or "gender nonconforming" or "nonbinary" or "agender*" or "bigender*" or "intergender*" or "inter-gender*" or "multigender" or "multi-gender*" or "genderqueer" or "gender queer" or "gender minority" or "gender minorities" or "gender divers*" or "gender incongruent" or "gender change" or "trans adult*" or "trans person*" or "trans male*" or "trans female*" or "cross gender" or "crossgender" or "exp Transvestism/" or "Transvest*" or "cross dress*" or "two-spirit*" or "gender affirm*" or "exp gender Dysphoria/" or "gender dysphor*" or "gender euphoria" or "gender identity disorder*" or "FTM" or "MTF" or "Male to female" or "Female to male").mp. 234670

2 ("exp Pharmacy/" or "exp Pharmacist/" or "exp Students, Pharmacy/" or "exp Education, Pharmacy/" or "exp Faculty, Pharmacy/" or "Pharmacy" or "Pharmacy student*" or "Pharmacist" or "Pharmacy curricul*" or "Pharmacy faculty" or "Pharmacy program*" or "Pharmacy education" or "Undergrad* pharmacy*" or "Bachelor of Science Pharmacy" or "BScPhm" or "Doctor of Pharmacy" or "PharmD").mp. 91118

3 1 and 2 426

**APA PsycInfo <1806 to November 2024 Week 2>**

1 ("exp Health Services for Transgender Persons/" or "exp Gender Identity/" or "Gender ident*" or "exp Transgender Persons/" or "transgender*" or "transperson*" or "transpeople*" or "trans gender*" or "exp Transsexualism/" or "trans patient" or "trans health*" or "trans* affirm" or "transexual*" or "trans sexual*" or "transman" or "transmen" or "trans man" or "trans men" or "transwoman" or "transwomen" or "trans woman" or "trans women" or "trans masc*" or "transmasc*" or "trans fem*" or "transfem*" or "gender quest*" or "gender fluid*" or "genderfluid*" or "gender non-conforming" or "gender nonconforming" or “gender continuum” or "nonbinary" or "agender*" or "bigender*" or "intergender*" or "inter-gender*" or "multigender" or "multi-gender*" or "genderqueer" or "gender queer" or "gender minority" or "gender minorities" or "gender divers*" or "gender incongruent" or "gender change" or "trans adult*" or "trans person*" or "trans male*" or "trans female*" or "cross gender" or "crossgender" or "exp Transvestism/" or "Transvest*" or "cross dress*" or "two-spirit*" or "gender affirm*" or "exp gender Dysphoria/" or "gender dysphor*" or "gender euphoria" or "gender identity disorder*" or "FTM" or "MTF" or "Male to female" or "Female to male").mp. 119511

2 ("exp Pharmacy/" or "exp Pharmacist/" or "exp Students, Pharmacy/" or "exp Education, Pharmacy/" or "exp Faculty, Pharmacy/" or "Pharmacy" or "Pharmacy student*" or "Pharmacist" or "Pharmacy curricul*" or "Pharmacy faculty" or "Pharmacy program*" or "Pharmacy education" or "Undergrad* pharmacy*" or "Bachelor of Science Pharmacy" or "BScPhm" or "Doctor of Pharmacy" or "PharmD").mp. 6857

3 1 and 2 76

**Embase Classic+Embase <1947 to 2024 November 15>**

1 ("exp Health Services for Transgender Persons/" or "exp Gender Identity/" or "Gender ident*" or "exp Transgender Persons/" or "transgender*" or "transperson*" or "transpeople*" or "trans gender*" or "exp Transsexualism/" or "trans patient" or "trans health*" or "trans* affirm" or "transexual*" or "trans sexual*" or "transman" or "transmen" or "trans man" or "trans men" or "transwoman" or "transwomen" or "trans woman" or "trans women" or "trans masc*" or "transmasc*" or "trans fem*" or "transfem*" or "gender quest*" or "gender fluid*" or "genderfluid*" or "gender non-conforming" or "gender nonconforming" or “gender continuum” or "nonbinary" or "agender*" or "bigender*" or "intergender*" or "inter-gender*" or "multigender" or "multi-gender*" or "genderqueer" or "gender queer" or "gender minority" or "gender minorities" or "gender divers*" or "gender incongruent" or "gender change" or "trans adult*" or "trans person*" or "trans male*" or "trans female*" or "cross gender" or "crossgender" or "exp Transvestism/" or "Transvest*" or "cross dress*" or "two-spirit*" or "gender affirm*" or "exp gender Dysphoria/" or "gender dysphor*" or "gender euphoria" or "gender identity disorder*" or "FTM" or "MTF" or "Male to female" or "Female to male").mp. 345531

2 ("exp Pharmacy/" or "exp Pharmacist/" or "exp Students, Pharmacy/" or "exp Education, Pharmacy/" or "exp Faculty, Pharmacy/" or "Pharmacy" or "Pharmacy student*" or "Pharmacist" or "Pharmacy curricul*" or "Pharmacy faculty" or "Pharmacy program*" or "Pharmacy education" or "Undergrad* pharmacy*" or "Bachelor of Science Pharmacy" or "BScPhm" or "Doctor of Pharmacy" or "PharmD").mp. 237205

3 1 and 2 1295

**Scopus**

TITLE-ABS-KEY ( "transgender*" OR "transperson*" OR "transpeople*" OR "trans gender*" OR "trans patient" OR "trans health*" OR "trans* affirm" OR "transexual*" OR "trans sexual*" OR "trans man" OR "trans men" OR "trans woman" OR "trans women" OR "trans masc*" OR "transmasc*" OR "trans fem*" OR "transfem*" OR "gender quest*" OR "gender fluid*" OR "genderfluid*" OR "gender non-conforming" OR "gender nonconforming" OR “gender continuum” OR "agender*" OR "bigender*" OR "intergender*" OR "inter-gender*" OR "multi-gender*" OR "gender queer" OR "gender minority" OR "gender minorities" OR "gender divers*" OR "gender incongruent" OR "gender change" OR "trans adult*" OR "trans person*" OR "trans male*" OR "trans female*" OR "cross gender" OR "Transvest*" OR "cross dress*" OR "two-spirit*" OR "gender affirm*" OR "exp gender Dysphoria/" OR "gender dysphor*" OR "gender euphoria" OR "gender identity disorder*" OR "FTM" OR "MTF" ) AND TITLE-ABS-KEY ( "Pharmacy" OR "Pharmacy student*" OR "Pharmacist" OR "Pharmacy curricul*" OR "Pharmacy faculty" OR "Pharmacy program*" OR "Pharmacy education" OR "Undergrad* pharmacy*" )

312 articles found
