# Supplementary material for: Gender-affirming care education in pharmacy: a scoping review protocol of practices in Canada and the USA
Source: BMJ Open. 2025 Jan 2;15(1):e086927. doi: 10.1136/bmjopen-2024-086927 (PMC11748774; doi:10.1136/bmjopen-2024-086927)
Supplement: Supplementary file 2 [file bmjopen-2024-086927.reviewer_comments.pdf]

PEER REVIEW HISTORY

BMJ Open publishes all reviews undertaken for accepted manuscripts. Reviewers are asked to complete a checklist review form and are provided with free text boxes to elaborate on their assessment. These free text comments are reproduced below.

ARTICLE DETAILS

Title (Provisional)

Title: Gender-Affirming Care Education in Pharmacy: A Scoping Review Protocol of Practices in Canada and the United States

Authors

Jackman, Liam; Kamran, Rakhshan

VERSION 1 - REVIEW

**Reviewer**	**1**
**Name**	**Glass, Beverley**
**Affiliation**	**James Cook University, Pharmacy**
**Date**	**13-May-2024**
**COI**	**Agreed - no competing interests**

Comments below:

1. In the abstract author refer to Canda and the United States - this should be reflected in the title.

2. This should also be reflected in the strengths and weaknesses - page 5 lines 96-102 as for example it is not comprehensive and in lines 97-98 studies from other countries are excluded.

3. There are a lot of secondary research questions (can they be refined and reduced) but none reflect the content and delivery of transgender education, which is referred to later in the protocol Maybe clarify what is meant by resources in line 215.

4. In the selection of the studies there is use of independent reviewers - are the authors planning to use consensus at any stage?

5. Relate lines 280-283 to the secondary questions - or reflect on the absence of this information in the secondary questions - as per comment 3. First time educational content and deliver is mentioned is on line 343

6. What is meant in line 310 by "the educational strategies will be summarized"

7. With the involvement Patients and the Public - explain why Ethics approval is not required or some acknowledgement to the participation of this group ethically.

8. In line 346 -comments is made about accreditation bodies - what about professional bodies offering CPD?

**Reviewer**	**2**
**Name**	**Crawford, Jess**
**Affiliation**	**University of Manitoba Faculty of Health Sciences, College of Nursing**
**Date**	**20-May-2024**
**COI**	**N/a**

20-May-2024

Dear Editorial Office,

Thank you for inviting me to review bmjopen-2024-086927 Gender-Affirming Care Education in Pharmacy: A Scoping Review Protocol.

I encourage you to review my comments to the authors, as attached to the review but wanted to connect regarding my selection of "no" to the question of *To the best of your knowledge is the paper free from concerns over publication ethics (e.g. plagiarism, redundant publication, undeclared conflicts of interest)?* I present the following:

You will notice in my comments that I made suggestions to look at our article (Crawford et al., 2023), but there are several places in the article that look like what we wrote in our protocol paper. While I recognize that there are only so many ways to write an introduction on the same topic, it raised a flag for me and so I wanted to flag this for you. In the table below are the areas I recognized

**Reviewed paper: bmjopen-2024-086927**	**Our paper:** Crawford, J., Schultz, A. S. H., Linton, J., Kramer, M., & Ristock, J. (2023). Gender-affirming care in undergraduate nursing education: A scoping review protocol. BMJ Open 13(3). https://bmjopen-bmj-com.uml.idm.oclc.org/content/bmjopen/13/3/e070576.full.pdf

"Gender affirming care also includes an awareness of and respect for transgender and non binary people, which means asking preferred name and pronouns, adopting gender neutral language and using a bioanatomy based approach to assessment and treatment[4]."	"Within nursing practice, this includes the universal adaptation of gender-neutral language and mitigating assumptions, such as by normalising using an organ and bioanatomy inventory during assessments and asking patients their chosen name and pronouns."
"Canada is the first country to collect data on gender diversity from a national<br>census. The most recent national census identified over 100 000 transgender or non binary individuals, with a higher proportion (1 in 150) among Canadians aged 15-34, and a lower proportion (1 in 550) among Canadians over the age of 35…"	"In Canada, over 100800 people were identified as TGD during the first collection of the national census data in 2021, and this number likely under-represents the population of TGD people."
"We do not know the precise number of providers delivering, or people receiving<br>gender affirming care in Canada, but we do know that access to gender-affirming care offers benefits…"	"While the number of people receiving, or providers delivering, gender-affirming care is unknown, we know that gender affirming are fosters positive health outcomes for TGD people"
Search strategy	Search strategy

Be well,

Jess Crawford, RN (they/them)

jess.crawford@umanitoba.ca

Reviewer Feedback for Gender-Affirming Care Education in Pharmacy: A Scoping Review Protocol

Comments to the Author:

> Thank you for the opportunity to review this important and timely study protocol. I really appreciated seeing the expansion of gender-affirming care education knowledge for another health profession, but I think there is a richer argument to be made here and some really important literature that is not being considered. I have suggested major revisions to ensure the affirming, safe nature of this work.

To position myself, I am a white, trans, non-binary academic who has published a couple of papers on gender affirming care in nursing education. I acknowledge that Dr. Annette Schultz, a white queer woman, supported me in reviewing your article. Below, we share some major suggestions for revisions with compassion and in hopes to ground this work in a gender affirming way. The general comments section elaborates on specific sections of the paper.

Major suggestions

From my postionality as a non-binary, trans person, as I read through the introduction of this paper, I found the rationale for this work was limited and somewhat offensive. Gender affirming care is beneficial and imperative for so many reasons beyond gender dysphoria. I invite you to consider a bigger, richer rationale for why this work is important. Concomitant is a shifting of lens to culturally safe care. As you mention, you bring a lens of cultural sensitivity, but this is an outdated, insufficient lens to navigate working towards equity. Please begin to shift to cultural humility and safety to formulate transparent, reciprocal, holistic, humble, and meaningful approaches to this work. You will need to consider more deeply how you will navigate literature that includes transgender people under variations of the 2SLGBTQ+ acronym. To address these considerations, please intentionally draw on the growing body of literature and reference accordingly. Further, while I applaud you for including patient/public involvement, there lacks transparency of (1) who are you, as authors, (2) who are partners involved (are they binary trans? Non-binary? cisgender? White?), (3) how the researchers are engaging them (i.e., one consult; did they review this manuscript), (4) how the patient/public partners are being compensated for their time/knowledge/lived experience (i.e., co-authorship, financial).

General comments

1. Abstract
    a. Great, concise abstract.
    b. Please consider adding Levac in the methods and mentioning patient/public involvement.
    c. “competence and confidence of future and current pharmacists in providing culturally sensitive care to transgender and gender diverse individuals” Reword according to culturally safe care (see point 5b).
2. Keywords:
    a. Consider *gender identity* as a keyword
3. Introduction
    a. The intro raises some good points but there's more development needed for both the logic and argument, as well as you should look at the current literature and bring it in to strengthen your rationale.
    b. While bioanatomy is certainly important, many (if not most) readers will be unclear as to what bioanatomy is. I suggest defining it in brackets.
    c. *Emerging* is said 2x in this line “The emerging social and political recognition of transgender and gender diverse people highlights the emerging need for gender-affirming care.”

d. Benefits of gender-affirming care span well beyond bolstering mental health. Your focus on *medicalized problems* that transgender and gender diverse people face is a deficit approach rather than an affirming approach to this work. I suggest drawing on emerging literature to really drive home why this work is needed, holistically. I can recommend looking to two of my publications, including looking at their references
   - Crawford, J., Schultz, A. S. H., Linton, J., Kramer, M., & Ristock, J. (2023). Gender-affirming care in undergraduate nursing education: A scoping review protocol. BMJ Open 13(3). https://bmjopen-bmj-com.uml.idm.oclc.org/content/bmjopen/13/3/e070576.full.pdf
   - Crawford, J., Schultz, A., & Chernomas, W. (2023) Interpersonal transphobia within nursing: A critical concept exploration. Advances in Nursing Science 47(2): 136-152. doi: 10.1097/ANS.0000000000000491

e. Preferred name/pronouns suggests that chosen pronouns are optional. Please change "preferred name and pronouns" to be more affirming.

f. Re: stats of trans folks in Canada, I am unclear the logic of the difference in ages and reporting and how that results in a generational gap. Please reference this and develop these ideas further.

g. Suggest to put in brackets "man or woman" to highlight what the *gender binary* is.

h. In my research, education and bias do not *overlook* trans people, but silences and erases them. Please consider shifting the language that suggests *bias* means *overlooking* people, with appropriate an reference.

i. Please expand on what you mean by "future developments" in the purpose statement.

j. "The findings from this scoping review will serve as" please move suggestions of findings to the implications section.

4. Methods
   a. Please join the 3 paragraphs into 1 paragraph.
   b. As authors, you hold power and bias. It is good practice to be transparent and state your positionality. Tell the reader who is involved and this allows the reader to critically reflect on what may be missing. Again, I direct you to the publications listed above, which each have positionality statements.
5. Stage 1:
   a. you use past tense, suggesting the study has already finished. Please address this and ensure the study is planned or ongoing.
   b. You outline the objective as *cultural sensitivity.* This practice is outdated and insufficient to address health and health equity for any population. Please look into *cultural humility* and/or *cultural safety,* related concepts which embrace life-long learning AND recognize that people cannot be "culturally competent" in a culture or know all of

cultures. Suggest looking at Foronda's work. And change throughout the paper.

6. Stage 2:
    a. "The search strategy was developed in consultation with the research team" - Please be transparent: who is part of the research team? Who helped develop this? Was a librarian involved?
    b. Please reference the "carefully chosen gender-diverse literature" where you drew keywords and subject headings from.
7. Stage 2, population section:
    a. Switch the period to a comma after pharmacists.
    b. Outline graduate vs. undergraduate education
    c. What about employee trainings, is this considered continuing education?
8. Stage 2, context section:
    a. Please outline how you intend to manage articles that look at LGBT+ which include transgender content?
    b. Please clarify if you plan to include reviews or primary studies only?
9. Stage 3:
    a. How will you account or address articles that look at the broader LGBT+ population, with some specificity to transgender?
10. Stage 4: Consider the following when charting the data:
    a. Who the population is (addressing secondary Q#1)
    b. Please report on student/faculty perceptions? (Related to secondary Q#4+5)
    c. Article recommendations/ implications are also needed as part of charting the data.
11. Stage 5:
    a. "The findings will be presented in a tabular format" starts the first 2 sentences in the first paragraph.
12. Patient and public involvement:
    a. Tell us more about the involvement - did they get to review this paper? What did you do? How many times did you meet? How will they be involved in decisions?
    b. Suggest looking to International public participation 2 guidelines https://cdn.ymaws.com/www.iap2.org/resource/resmgr/pillars/Spectrum_8.5x11_Print.pdf
    c. Transparency: please consider stating if/how this group is compensated for their time, expertise, and/or lived experience. If this group is not compensated, consider other forms of reciprocity (i.e., co-author recognition)
13. Conclusion:
    a. Please offer a conclusion paragraph to the reader.
14. Search strategy:
    a. Consider adding *gender continuum* or *trans patient* or *trans health** or *trans* affirm*, Subject heading: Health services for Transgender persons
15. PRISMA

a. Consider registering this protocol with OSF.
b. You highlight that critical appraisal will be done in PRISMA but not in the paper, please address this discrepancy.

VERSION 1 - AUTHOR RESPONSE

Reviewer 1		
Comment	Response	Change to the Manuscript
In the abstract author refer to Canda and the United States - this should be reflected in the title.	Thank you for this comment – we agree and have adjusted the title accordingly.	Title: Gender-Affirming Care Education in Pharmacy: A Scoping Review Protocol of Practices in Canada and the United States
This should also be reflected in the strengths and weaknesses - page 5 lines 96-102 as for example it is not comprehensive and in lines 97-98 studies from other countries are excluded.	Thank you for this comment. We have detailed this exclusion criteria in the strengths and weaknesses section and exclusion criteria.	Change to strengths and weaknesses section:<br><br>- Articles eligible for inclusion in this scoping review are limited to English and French and published in Canada or the United States; therefore, it is possible that relevant studies in other languages/countries may be missed.<br><br>Change to exclusion criteria:<br><br>- Study only includes articles from Canada or the United States
There are a lot of secondary research questions (can they be refined and reduced) but none reflect the content and delivery of transgender education, which is referred to later in the protocol Maybe clarify what is meant by resources in line 215.	Thank you for this comment. We have reduced the quantity of secondary research questions and included a question about the content and delivery of transgender education. We have also clarified what is meant by resources.	Reduced quantity of secondary research questions, and added:<br><br>• What is the content within gender-affirming care education, and how is the education delivered?

		Specification of what is meant by resources: "…educational resources – such as webinars, online modules, or online pamphlets)."
In the selection of the studies there is use of independent reviewers - are the authors planning to use consensus at any stage?	Thank you for this comment, yes, the aim is for consensus to be reached by two independent reviewers. If consensus cannot be reached, a third reviewer will resolve discrepencies.	Clarification of the use of consensus:<br><br>"The titles and abstracts will be screened by two independent reviewers, with an aim to reach consensus. The reviewers will pilot test 20 titles and abstracts and then attend a meeting to discuss discrepancies before proceeding further. If consensus cannot be reached, discrepancies will be resolved by a third independent reviewer. Similarly, the full text documents will subsequently be screened by two independent reviewers. The discrepancies will again be resolved by a third independent reviewer."
Relate lines 280-283 to the secondary questions - or reflect on the absence of this information in the secondary questions - as per comment 3. First time educational content and deliver is mentioned is on line 343	Thank you for this comment – we have modified the secondary questions to reflect lines 280-283 and 343.	Secondary questions amended to reflect the rest of the paper.
What is meant in line 310 by "the educational strategies will be summarized"	Thank you for this comment. We have removed this sentence as it is unclear, and we meant to describe details of the narrative synthesis, which is already	Clarification provided about narrative synthesis, removal of unclear sentence:<br><br>"The results will be conveyed through narrative synthesis, delving into educational content and

	described in the rest of the paragraph.	education delivery with an examination of its impact on pharmacy practice and patient care. The use of narrative synthesis allows for complex information to be synthesized into a comprehensive narrative that captures the core of the researching findings but does find a limitation in that it is inherently subjective, relying in individual interpretation of the data, and therefore can lack transparency. To address this limitation, we will adhere to the synthesis without meta-analysis (SWiM) guideline to ensure systematic and evidence-based reporting."
. With the involvement Patients and the Public - explain why Ethics approval is not required or some acknowledgement to the participation of this group ethically.	Thank you for this comment. Our patient and public partners are not research subjects, they are partners in the research and will be acknowledged as co-authors on the paper or in the acknowledgement section of the paper, given their level of involvement and if they meet ICJME criteria.	Clarification of the role of patient partners, and specifying that our partners are not research subjects.<br><br>"Patient and public partners will be present as co-authors or mentioned in the acknowledgment section of the final paper, given level of involvement and if they meet authorship criteria. Patient and public partners are not research subjects, they are part of the research team."
In line 346 -comments is made about accreditation bodies - what about professional bodies offering CPD?	Thank you for this comment. We have included additional information about professional bodies offering CPD.	Clarification on how the work relates to CPD bodies:<br><br>"The findings of this scoping review aim to inform pharmacy students, pharmacists, pharmacy educators, educational institutions, healthcare policymakers, accreditation bodies, and professional

		bodies offering continuing professional development (CPD) about gender diversity and gender-affirming care”

Reviewer 2		
Comment	Response	Change to the Manuscript
Major Suggestions		
Gender affirming care is beneficial and imperative for so many reasons beyond gender dysphoria. I invite you to consider a bigger, richer rationale for why this work is important.	Thank you for this comment – we appreciate this insight. We have added statements that show that gender-affirming does more than affirm gender and alleviate gender-dysphoria but also augments wellbeing.	Additional statements included:<br><br>“Gender affirming care affirms one’s gender, alleviates gender dysphoria, and augment wellbeing. Gender-affirming care promotes positive identify development, self-esteem, and social participation, supporting individuals in living true to themselves and forming meaningful connections within their communities. Gender-affirming care also results in reductions an anxiety, depression, and suicidality.”
Concomitant is a shifting of lens to culturally safe care. As you mention, you bring a lens of cultural sensitivity, but this is an outdated, insufficient lens to navigate working towards equity. Please begin to shift to cultural humility and safety	Thank you for this comment – we agree with this point and appreciate that you brought it to our attention. We have added an additional section to address this in the introduction.	Additional section included:<br><br>“In addition, pharmacy curricula must move one step further: not only teaching what care should be delivered

to formulate transparent, reciprocal, holistic, humble, and meaningful approaches to this work.		but also how to deliver that care in a way that is patient-centered and responsive to the needs to transgender and gender-diverse individuals. This requires integrating frameworks of cultural sensitivity, cultural humility, and cultural safety. Cultural sensitivity serves as a foundation, by teaching students about the use of inclusive language, ensuring that they develop the practical skills needed to show respect for diverse identities. Cultural humility builds on this by providing students with opportunities for self-reflection, teaching them about the identification of implicit biases, and promoting lifelong learning. Cultural safety build on this again by addressing systemic inequities in healthcare, teaching students to critically think about and challenge structural barriers that contribute to health inequities."
You will need to consider more deeply how you will navigate literature that includes transgender people	Thank you for this comment. We will be included articles that focus on transgender and gender diverse	Clarification added on how we will navigate literature that includes

under variations of the 2SLGBTQ+ acronym. To address these considerations, please intentionally draw on the growing body of literature and reference accordingly.	individuals, or where information exists which can be analysed through a sub-group analysis if the study sample includes a relevant sub-group which is transgender or gender-diverse. We have referenced two prior reviews from our team where we have handled this, including an article published in JAMA Open on global gender-affirming care, and Paediatrics and Child Health, the official journal of the Canadian Paediatrics Society. Our research team has a documented and published history of systematic reviews in gender-affirming care where we have addressed these considerations.	includes transgender people under variations of the 2SLGBTQ+ acronym. We have cited examples of past systematic reviews on gender-affirming care published from our team in high-impact journals as a reference:<br><br>Our search strategy includes terms related to the 2SLGBTQ+ acronym, however, the focus of this scoping review is on transgender and gender diverse populations and gender-affirming care. Articles will be carefully screened in line with the above inclusion/exclusion criteria to include studies which focus on gender-affirming care for transgender or gender diverse populations, or where a relevant subgroup is clearly defined that can be extracted from. Our team has successfully considered these concepts in the past with two high-impact systematic reviews on international gender-affirming care [JAMA O], and Canadian paediatric

		gender-affirming care [peds paper].
Further, while I applaud you for including patient/public involvement, there lacks transparency of (1) who are you, as authors, (2) who are partners involved (are they binary trans? Non-binary? cisgender? White?), (3) how the researchers are engaging them (i.e., one consult; did they review this manuscript), (4) how the patient/public partners are being compensated for their time/knowledge/lived experience (i.e., co-authorship, financial).	Thank you for this comment. We agree that PPI is essential for all work in this area, and we have included further clarification in this section in line with your points.	Additional clarification added in the PPI section:<br><br>"The authors of the scoping review consist of cisgender and nonbinary individuals, including individuals who are members of the 2SLGBTQ+ community. All of the authors hold higher-level clinical or research degrees (e.g., PhD, MD, PharmD, MSc) and are clinician-scientists. Seven patient and public partners are also present on the research team. These include three trans women, two trans men, and two non-binary individuals. Patient and public partners are predominantly White (6/7). Patient and public partners were recruited through contacting transgender charities (i.e., Gender Identity Research and Education Society), online community transgender support groups, and recruitment of one individual from an existing patient group with a

		separate research team. Patient and public partners are involved with confirming relevance and importance of the research questions, reviewing the search strategy, and reviewing the final results of the scoping review and manuscript. Patient and public partners will be present as co-authors or mentioned in the acknowledgment section of the final paper, given level of involvement and if they meet authorship criteria. Patient and public partners are not research subjects, they are part of the research team."
Abstract		
Please consider adding Levac in the methods and mentioning patient/public involvement.	Thank you for this comment. We have mentioned Levac in the methods and mentioned PPI in the methods as well.	Inclusion of Levac and PPI in the methods.
"competence and confidence of future and current pharmacists in providing culturally sensitive care to transgender and gender diverse individuals" Reword according to culturally safe care (see point 5b).	Thank you for this comment. Per the editor comment, this sentence was removed.	Removal of sentence per editor comment.
Introduction		
The intro raises some good points but there's more development needed for both the logic and argument, as well as you should look at the current literature and	Thank you for this comment. We have made some significant changes to the introduction to strengthen our rationale – many of these	Re-written introduction.

bring it in to strengthen your rationale.	changes are highlighted via comments below.	
While bioanatomy is certainly important, many (if not most) readers will be unclear as to what bioanatomy is. I suggest defining it in brackets.	Thank you for this comment – we agree and have adjusted accordingly.	Additional information included:<br><br>"Gender-affirming care also includes practices that demonstrate awareness of and respect for different gender identities, including asking about and using a person's name and pronouns, adopting gender-neutral language, and using a bioanatomy based approach (which considers a person's anatomy without making assumptions about gender identity)"
Emerging is said 2x in this line "The emerging social and political recognition of transgender and gender diverse people highlights the emerging need for gender-affirming care."	Thank you for this comment – We reviewed and determined that including the ages/ suggesting that there is a generational gap did not add meaningfully to the manuscript. We simplified the statistics to highlight the total number of transgender individuals in Canada, focusing on clarity and relevance of included information. We therefore were able to remove this statement altogether.	Rewritten introduction to improve clarity of this sentence.
Benefits of gender-affirming care span well beyond bolstering mental health. Your focus on medicalized problems that transgender and gender diverse people face is a deficit approach rather than an affirming	Thank you for this comment – we appreciate this insight. We have added statements that show that gender-affirming does more than affirm gender and alleviate gender-dysphoria but also augments wellbeing.	Additional information included:<br><br>"Gender affirming care affirms one's gender, alleviates gender dysphoria,

approach to this work. I suggest drawing on emerging literature to really drive home why this work is needed, holistically. I can recommend looking to two of my publications, including looking at their references		and augment wellbeing. Gender-affirming care promotes positive identify development, self-esteem, and social participation, supporting individuals in living true to themselves and forming meaningful connections within their communities. Gender-affirming care also results in reductions an anxiety, depression, and suicidality.”
Preferred name/pronouns suggests that chosen pronouns are optional. Please change “preferred name and pronouns” to be more affirming.	Thank you for this comment – we agree. We have removed the word “preferred”.	Sentence amended:<br><br>“Gender-affirming care also includes practices that demonstrate awareness of and respect for different gender identities, including asking about and using a person’s name and pronouns, adopting gender-neutral language, and using a bioanatomy based approach.”
Re: stats of trans folks in Canada, I am unclear the logic of the difference in ages and reporting and how that results in a generational gap. Please reference this and develop these ideas further.	Thank you for your comment. We reviewed and determined that including the ages/ suggesting that there is a generational gap did not add meaningfully to the manuscript. We simplified the statistics to highlight the total number of transgender individuals in Canada, focusing on clarity and	In Canada, the most recent national census identified over 100,000 transgender and non-binary individuals, representing a significant number of people who would benefit from

	relevance of included information.	access to gender-affirming care.
Suggest to put in brackets “man or woman” to highlight what the gender binary is.	Thank you for this comment, we agree and have adjusted accordingly.	Amended sentence:<br><br>“Health inequities: disparities in the distribution of health resources and health status, may be attributed in part to cisnormativity in healthcare, the assumption that people will identify with the gender binary (male or female)”
In my research, education and bias do not *overlook* trans people, but silences and erases them. Please consider shifting the language that suggests *bias* means *overlooking* people, with appropriate an reference.	Thank you for this comment. We have adjusted accordingly and added a reference.	Amended sentence:<br><br>“This bias is perpetuated by educational curricula that excludes or even erases the needs of transgender and non-binary individuals, ultimately resulting in healthcare professionals feeling unprepared to care for this population.”
Please expand on what you mean by “future developments” in the purpose statement.	Thank you for this comment – we agree that “future developments” was not specific. We have removed this statement from the introduction, we felt it would be more appropriately addressed in the implications section.	The statement was removed was removed from the introduction.
“The findings from this scoping review will serve as” please move suggestions of findings to the implications section.	Thank you for this comment – we agree that it is better in the implications section, therefore, have removed it from the introduction.	The statement was removed from the introduction.
Methods		

Please join the 3 paragraphs into 1 paragraph.	Thank you for this comment. We have joined the 3 paragraphs into 1 paragraph.	Three paragraphs joined into 1 paragraph.
As authors, you hold power and bias. It is good practice to be transparent and state your positionality. Tell the reader who is involved and this allowsthe reader to critically reflect on what may be missing. Again, I direct you to the publications listed above, which each have positionality statements.	Thank you for this comment. We have included a positionality section.	Positionality section included:<br><br>"The authors of the scoping review consist of cisgender and nonbinary individuals, including individuals who are members of the 2SLGBTQ+ community. All of the authors hold higher-level doctoral degrees (e.g., PhD, MD, PharmD) and are clinician-scientists working at the University of Toronto or the University of Oxford. The authors are published researchers in the field of gender-affirming care, with the senior author having completed their Oxford doctoral thesis in the area."
Stage 1:<br>1. you use past tense, suggesting the study has already finished. Pleaseaddress this and ensure the study is planned or ongoing.<br>2. You outline the objective as *cultural sensitivity.* This practice is	Thank you for these comments:<br>1. Thank you for catching this. The study is indeed ongoing. We have amended sentence tense.<br>2. Thank you for this comment, we have made this change throughout the paper to remove cultural sensitivity and instead have used the terms culturally safe or cultural humility	Amended tense in Stage 1, removed use of cultural safety throughout manuscript.

outdated and insufficient to address health and health equity for any population. Please look into *cultural humility* and/or *cultural safety,* related concepts which embrace life-long learning AND recognize that people cannot be “culturally competent” in a culture or know all of cultures. Suggest lookingat Foronda’s work. And change throughout the paper.		
Stage 2:<br>1. “The search strategy was developed in consultation with the research team” - Please be transparent: who is part of the research team? Who helped develop this? Was a librarian involved?<br>2. Please reference the “carefully chosen gender-diverse literature” where you drew keywords and subject headings from.	1. Thank you for this comment. We have clarified that a health sciences librarian was involved. The senior author and lead author developed the search strategy with input from PPI.<br>2. Thank you for this comment. We have included these citations.<br>3. Period switched to a comma.<br>4. Inclusion of graduate vs undergraduate	Amendments made in Stage 2 to clarify the search strategy and inclusion criteria.

Stage 2, population section:<br>1. Switch the period to a comma after pharmacists.<br>2. Outline graduate vs. undergraduate education<br>3. What about employee trainings, is this considered continuing education?	education added<br>5. Yes – we have clarified that this could also represent employee trainings and have clarified this	
Stage 2, context section:<br>1. Please outline how you intend to manage articles that look at LGBT+which include transgender content?<br>2. Please clarify if you plan to include reviews or primary studies only?	Thank you for these comments. We have clarified that we are focusing on primary articles and how we will focus on articles that focus on transgender and gender diverse individuals or where a relevant identified subgroup is available. We have discussed this earlier in the text in detail with citations to previous work.	Clarifications on focus on primary articles, and clarification of study population.
Stage 3:<br>1. How will you account or address articles that look at the broader LGBT+ population, with some specificity to transgender?	Thank you for this comment. We have specified this above, and earlier in the paper as well with reference to our JAMA paper and other past published systematic reviews from our team.	Clarification added earlier in the text regarding this point, with inclusion of relevant citations and scholarships which evidence how this has successfully been handled in the past.
Stage 4: Consider the following when charting the data:<br>1. Who the population is (addressing secondary Q#1)<br>2. Please report on student/faculty perceptions?	Thank you for this comment. We have included these as items for data extraction.	Items suggested by reviewer included as data items for extraction.

(Related to secondary Q#4+5)<br>3. Article recommendations/ implications are also needed as part of chartingthe data.		
Stage 5:<br>1. "The findings will be presented in a tabular format" starts the first 2 sentences in the first paragraph.	Thank you for this comment. We have removed this repetition.	Sentence repetition removed.
Patient and public involvement:		
1. Tell us more about the involvement - did they get to review this paper?What did you do? How many times did you meet? How will they be involved in decisions?<br>2. Transparency: please consider stating if/how this group is compensatedfor their time, expertise, and/or lived experience. If this group is not compensated, consider other forms of reciprocity (i.e., co-author recognition)	Thank you for this comment. We have clarified the patient and public involvement further.	Re-written PPI section:<br><br>"Seven patient and public partners are also present on the research team. These include three trans women, two trans men, and two non-binary individuals. Patient and public partners are predominantly White (6/7). Patient and public partners were recruited through contacting transgender charities (i.e., Gender Identity Research and Education Society), online community transgender support groups, and recruitment of one

		individual from an existing patient group with a separate research team. Patient and public partners are involved with confirming relevance and importance of the research questions, reviewing the search strategy, and reviewing the final results of the scoping review and manuscript. Patient and public partners will be present as co-authors or mentioned in the acknowledgment section of the final paper, given level of involvement and if they meet authorship criteria. Patient and public partners are not research subjects, they are part of the research team. Patient and public partners and research team members met over Microsoft Teams, where a meeting agenda was circulated prior to online meetings. An average of 2-3 meetings are held per year with regular email communication in the interim for the current research project, as well as others that patient

		and public partners are involved with. During meetings, disagreements between PPI are resolved through discussion until all participants reach consensus."
Conclusion:<br>1. Please offer a conclusion paragraph to the reader.	Thank you for this comment. We have included a conclusion paragraph:	Conclusion paragraph added:<br><br>"In conclusion, this scoping review protocol outlines a research study which will be conducted to better understand gender-affirming care education in pharmacy. The results of this research can be used by clinicians, policymakers, educators, and commissioners to better identify gender-affirming education in pharmacy, and understand important gaps for improvement. The results from this study will also complement other important gender-affirming care education research conducted across various clinical areas, to ensure comprehensive and holistic progress in gender-affirming care education across various

		clinical professions and settings.”
Search strategy:		
1. Consider adding *gender continuum* or *trans patient* or *trans health** or *trans* affirm*, Subject heading: Health services for Transgender persons	Thank you for this comment. We have included these terms in the search strategy.	Search strategy amended to included recommended terms.
PRISMA<br>1. Consider registering this protocol with OSF.<br>2. You highlight that critical appraisal will be done in PRISMA but not in thepaper, please address this discrepancy.	1. Thank you for these comments. We will consider registration of this protocol in OSF once it has been peer-reviewed to ensure the OSF version reflects the final version.<br>2. Thank you for this comment. PRISMA is a reporting guideline, and not a critical appraisal tool. We have clarified this in our paper.	Clarification that PRISMA is a reporting guideline and that the review will follow the PRISMA guideline for reporting:<br><br>“The scoping review will be reported following the Preferred Reporting Items for Systematic Reviews and Meta-Analyses extension for scoping reviews (PRISMA-ScR)[12].”
